# The potential of postharvest zinc treatment for preservation of pomegranate aril quality

**DOI:** 10.1038/s41598-024-51437-5

**Published:** 2024-01-11

**Authors:** Reihaneh Aminzade, Asghar Ramezanian, Saeid Eshghi, Seyed Mohammad Hashem Hosseini

**Affiliations:** 1 Department of Horticultural Science, School of Agriculture, Shiraz University, Shiraz, Iran; 2 Department of Food Science and Technology, School of Agriculture, Shiraz University, Shiraz, Iran

**Keywords:** Physiology, Plant sciences

## Abstract

A short shelf life usually limits the distribution and supply of pomegranate arils. Since zinc (Zn) has an indispensable role in the nutrient integrity of our diet and is effective in suppressing pathogens, this study was done as two separate experiments of pre-harvest spraying and postharvest dipping of arils with two zinc supplements, including nano zinc oxide (nZnO) and zinc sulfate (ZnSO_4_). The optimized concentration of both sources was used in the experiment. The pre-harvest treatment failed to extend the shelf life of arils, and, ultimately, the arils decayed after 15 days. However, the postharvest zinc treatment significantly (P < 0.01) affected all measured indices. Also, zinc sulfate was more effective than nZnO. Zn uptake was higher in postharvest treatments because exogenous Zn was in direct contact with the aril surface. After dissolving in water, Zn ions in sulfate bind to the membrane of microorganisms and thus delay cell division and microbial growth cycle. The solubility of zinc oxide nanoparticles in water is poor. Using the ZnSO_4_ treatment (0.8%W/V) effectively maintained the values of titratable acidity (TA), total phenolic content (TPC), total soluble solids (TSS), anthocyanin content, and antioxidant activity. Also, this treatment significantly controlled weight loss in the arils.

## Introduction

Pomegranate (*Punica granatum* L.) is a prominent species in the *Lythraceae* family, with wide areas of distribution in arid and semi-arid climates^1^. Pomegranate arils contain valuable nutrients and bioactive compounds^2^. As a large source of phenolic substances and anthocyanins, arils contribute to the inhibition of free-radicals and reduce the chances of disease^3^.

Pomegranate arils are traditionally hand-peeled from pomegranate fruits, which is a laborious process and limits the extent of its consumption. With the advancement of technology, however, there is a great desire to use ready-to-use arils. Pomegranate arils usually have a short shelf life because of their thin protective layer that cannot effectively resist water loss and pathogenic penetration. Thus, the arils deteriorate rapidly during storage, causing problems in their distribution and supply^1^. Various methods were reportedly effective in increasing their shelf-life. These methods include, but are not limited to, modified atmosphere packaging, nano edible coatings^4^ and edible coatings^5^, essential oils^6^, and active packaging^7^. Chitosan coated ZnO nano-composites have shown antibacterial and antifungal activity^8^.

As an essential element, zinc is inseparable from the diets of many mammals, including humans. Recently, its bio fortification has been proposed as an efficient technique to meet human nutritional needs^9^. Zinc actively participates in the catalytic activity of over 300 enzymes while being a cofactor of superoxide-dismutase (SOD)^10^. Zinc deficiency affects the structure and function of cellular membranes by making them more susceptible to oxidative damage^11^. In humans, zinc-deficient diets can lead to retarded growth. This has prompted researchers to propose different ways for the incorporation of zinc into food, including diversification/modification, fortification, supplementation, and biofortification^12^.

Arils are increasingly becoming popular as a freshly-consumed option and in the formulation of desserts. Meanwhile, zinc-enriched arils may alleviate zinc deficiency in humans. Since zinc is reportedly known for antifungal activity^13,14^, using nano zinc oxide (nZnO) and carboxyl methyl cellulose (CMC) on arils can effectively increase aril shelf life^15^. Roohani et al. (2013)^12^ reported how zinc oxide can act against bacteria and various other pathogens. Zinc is an environmentally-friendly substance with low toxicity^9^.

The enrichment of fruits with essential elements after harvest is a new method of meeting human nutritional needs. Such enrichment efforts can reduce human diseases and deficiencies faster and more cost-effectively than pre-harvest methods, especially when used on agricultural products that can benefit from an increase in postharvest storability and the maintenance of fruit quality during storage^12^. A comprehensive review of the available literature revealed no considerable amount of previous research on using zinc for the objective of prolonging the shelf-life of horticultural produce. So far, no report has been published to elaborate on a comparative analysis of pre- and post-harvest zinc application on pomegranate arils. Accordingly, the aim of this research was to draw comparisons between two zinc sources (i.e. nZnO and ZnSO_4_) and their separate applications in pre- and post-harvest conditions. Evaluations were made on the qualitative characteristics of arils, microbial growth inhibition during cold storage, and the effects of zinc enrichment.

## Materials and methods

### Plant materials, fruit treatments and storage condition

The experiment started two months before the commercial harvest of pomegranates, as the qualitative features of pomegranate fruits began to change. The fruits were harvested from an orchard in Farooq (29° North and 53° East). The pomegranate cultivar was Shirin Shahvar, an important commercial cultivar in Iran. There were two stages of foliar application, including 2 months before harvest and one month before harvest. Zinc sulfate and nZnO were used as two separate zinc sources. Each source was applied at four concentrations (0, 0.4, 0.6 and 0.8%W/V) in three replications in preliminary experiments and the optimized concentration was applied in the main experiment. Pomegranates were harvested from mature trees in mid-September at the time of commercial harvest according to an appropriate TSS/TA (24–27) ratio. Healthy pomegranates were promptly taken to the lab for assessments. Pomegranates were immersed in NaOCl (0.02%) for 5 min before washing. The arils were organized into three replications, each weighing around 50 gr, and were packaged in polypropylene containers for the pre-harvest treatment. A number of healthy pomegranates from untreated trees were also transferred to the laboratory and disinfected using similar procedures. The arils were removed from the fruits accordingly. The arils were immersed in solutions of 0 (distilled water), 0.4, 0.6 and 0.8%W/V zinc sulfate, and nZnO for one minute. nZnO was purchased from the US-NANO company with a purity of 99.8%. The nZnO ranged in size, from 10 to 30 nm, which involved having a suspension of ultrasonic-assisted dispersion of nZnO in distilled-water^16^. When the treatments ended, the extra moisture was left to evaporate from the aril surface and then the arils were packed in polypropylene containers, similar to the pre harvest stage, but this was regarded as the postharvest treatment. All polypropylene containers remained in cold storage (5 °C, 95–90% RH) for 30 days. The arils were sampled on days 1, 5, 10, 15, 20, 25, and 30. Quantitative and qualitative assessments involved measuring several variables, namely, zinc content, the effective duration of storage, pH, weight loss (WL), titratable acidity (TA), total soluble solids (TSS), antioxidant activity, anthocyanin content, TPC, microbial contamination, and polyphenol oxidase (PPO) activity. Trained panelists were asked to evaluate the sensory qualities of arils when the storage period ended. Any sign of mold growth meant the end of storage life.

### Physicochemical assessment qualitative factors

On days 1, 5, 10, 15, 20, 25, and 30 of cold storage, quantitative and qualitative data were evaluated. After removing the arils from storage, they were kept at 25 °C for 6 h before the assessments.

### Zinc content

The Zn content was measured by an atomic absorption spectrometer (Shimadzu Model 650AA, Japan) to determine the zinc content of all arils. The results were expressed as mg/kg fresh weight (FW).

### Storage life

The quality of arils was observed during storage to determine the occurrence of mold growth. The storage life of arils was calculated as the amount of time between packaging and the appearance of initial mold growth. To determine the traits affecting shelf life, a stepwise regression analysis was used for the shelf life as a dependent variable and traits such as Zn, TPC, total aerobic microorganisms, psychrotrophic bacteria, total yeast and mold counts, TA, PPO and WL were entered into the model, respectively (Table 1).

**Table 1:** Stepwise regression analysis for shelf life and Path analysis of studied traits.

Variable	Estimated parameter	Partial R2	Model R2	F value			
Zn	0.86	0.91	0.91	394			
TPC	0.001	0.035	0.94	23.65			
Total aerobic count	1.29	0.022	0.96	23.48			
TA	18.04	0.00*9*	0.97	12.44			
Total yeast and mold	0.15	0.0056	0.98	9.67			
PPO	− 0.26	0.0017	0.98	3.11			
Psychrotrophic	− 0.62	0.002	0.98	3.97			
WL	5.92	0.0015	0.98	7.38			
Intercept	1.88						

Variable	Direct effect	Indirect effect					Correlation
		TA	TPC	Total aerobic count	Total yeast and mold	Zn	
TA	0.1604	–	0.0366	− 0.1157	− 0.01119	0.125	0.8498
TPC	0.1587	0.0362	–	0.0086	− 0.0011	− 0.0115	0.1165
Total aerobic count	0.2676	− 0.1932	0.0146	–	0.2443	− 0.2477	− 0.8283
Total yeast and mold	0.1954	− 0.1364	− 0.0014	0.1784	–	− 0.174	− 0.7962
Zn	1.2611	0.9828	− 0.0920	− 1.1673	− 1.1229	–	0.9527

### Weight loss

The WL of arils was a description of the difference between the initial weight of arils (W_1_) and their secondary weight at each sampling time (W_2_). Using Eq. (1), the WL was calculated^17^.

1 $${\text{Weight}}\,{\text{Loss}}\,\left( \% \right)\, = \,\left( {{\text{W}}_{{1}} - {\text{ W}}_{{2}} /{\text{ W}}_{{1}} } \right)\, \times \,{1}00 .$$

### TA, TSS, and juice pH

A pH meter was used for determining the pH of the juice (JENWAY 351, England). Calibrations were made by pH buffers of 7 and 4^18^. A manual refractometer (ATAGO, Japan) enabled the measurement of TSS in percentages. After titration with 3 mL, aril juice was examined for TA, using 0.1 mol/L NaOH for reaching pH 8.2 as the end of titration. The results were reported as citric acid (%)^15^.

2 $${\text{TA}}\, = \,{\text{N}}\, \times \,{\text{V}}\, \times \,{\text{E}}/{\text{D}}\, \times \,{1}00.$$

Organic acids (TA) were quantified as mg 100 m/L, where E, V, and N are the gram valence of the major organic acid (citric acid), the NaOH volume, and the normality of NaOH, respectively. Sample volume is D (ml). Ultimately, the organic acid content was expressed as mg/100 mL.

### Total phenolic content

TPC was evaluated based on the Folin-Ciocalteu colorimetric method^19^. For this purpose, 100 μl of the extract was combined with 100 μl of 2% sodium carbonate and left at room temperature for 3 min. The samples were then treated with 20 μl of 50% folin and stored at room temperature for 30 min. The mixture absorbance was measured using a microplate spectrophotometer at a wave length of 750 nm (Epoch Biotech, Germany). TPC was measured in mg GAE/L.

### Anthocyanin

The anthocyanin content was assessed by means of a spectrophotometer^19^. Two buffers were used for this purpose: sodium-acetate (0.4 M) for pH 4.5 and potassium-chloride (0.025 M) for pH 1.0 according to Eq. (3). The corresponding buffer (1:5) was applied for diluting each sample, followed by measuring the absorbance at 700 and 510 nm. The anthocyanin content was calculated via Eq. (3), resulting in values of cyanidin-3-glucoside equivalents (mg/L).

3 $${\text{A}}\, = \,\left( {{\text{A51}}0\, - \,{\text{A7}}00} \right)\,{\text{pH 1}}.0\, - \,\left( {{\text{A51}}0\, - \,{\text{A7}}00} \right)\,{\text{pH 4}}.{5} .$$

$${\text{Anthocyanin }}\left( {{\text{mg}}/{\text{L}}} \right) \, = {\text{A }} \times {\text{ MW }} \times {\text{ DF }} \times { 1}000/\varepsilon \, \times { 1},$$

where MW is the molecular weight (449.2 g/mol), A is the absorbance, ε is the molar absorptivity (26,900), and DF is the dilution factor.

### Antioxidant activity

The antioxidant activity of each extract was determined via DPPH free-radical scavenging^20^. Accordingly, the extract (100 mL) was diluted in 1 mL Tris buffer and 0.1 mM DPPH (pH = 7.5) (1 mL). Then, it was stored at 25 °C for 30 min and the absorbance was read at 517 nm. Ultimately, Eq. (4) was applied to calculate the activity of antioxidants.

4 $${\text{Antioxidant activity}}\left( \% \right) = { 1} - {\text{ A Sample }}\left( {\text{517 nm}} \right)/{\text{ A Control }}\left( {\text{517 nm}} \right) \, \times {1}00.$$

### Polyphenol oxidase activity

Extraction and relevant assays were performed according to Shelke et al. (2014)^21^ with minor modifications. Ten gr of aril juice was homogenized in phosphate buffer (20 mL, 0.05 M, pH 7.0). The resultant solution entered a centrifuge of 10,000 rpm (30 °C) for 30 min. For the enzyme assay, 500 μl of catechol reagent was combined with phosphate-buffer (2 ml, pH 7.0) and enzyme extract (500 μl). A wavelength of 420 nm was used for reading the sample (Spectronic, USA). The difference in absorbance (420 nm) per liter of fresh weight in each minute (U/L) determined the PPO activity.

### Microbiological evaluations

Three packages from each treatment were randomly chosen on each sampling time, and a series of ten-fold dilution sets were prepared in 0.8% NaCl (w/v). The pour-plate method was used for counting psychotrophic bacteria and total aerobic microorganisms after incubation at 7 °C in a week, or at 30 °C in 72 h, respectively. Violet-Red Bile-Lactose Agar (VRBLA, Sigma) plates and Yeast-Extract Glucose-Chloramphenicol agar (YGC agar, Sigma) were applied for measuring the total yeast content, total coliform counting, and mold population after incubation at ambient temperature in 5 days (37 °C). Each test was repeated and the colony’s population was determined as log_10_ cfu/g^22^.

### Sensory evaluation

The sensory features of arils were comprised of sweetness, sourness, luminosity, color, sliminess, and aroma. Ten individuals were asked to taste the samples, fifteen days after the cold-storage (5 °C). The desirability of each sample was scored from 1 to 10, scaling from least desirable to most desirable, by means of average values^23^.

### Statistical analyses

Each treatment group was placed in a factorial design, with RCBD in three replicates. Physicochemical values were analyzed and, after Duncan’s test (P ≤ 0.01), all data entered SAS (SAS Institute Inc., USA). Pearson’s correlation coefficients were described among the variables.

### Plant material statement

Plant material in this research comply with relevant institutional, national, and international guidelines and legislation.

## Results and discussion

### Zinc content

**Figure 1:**
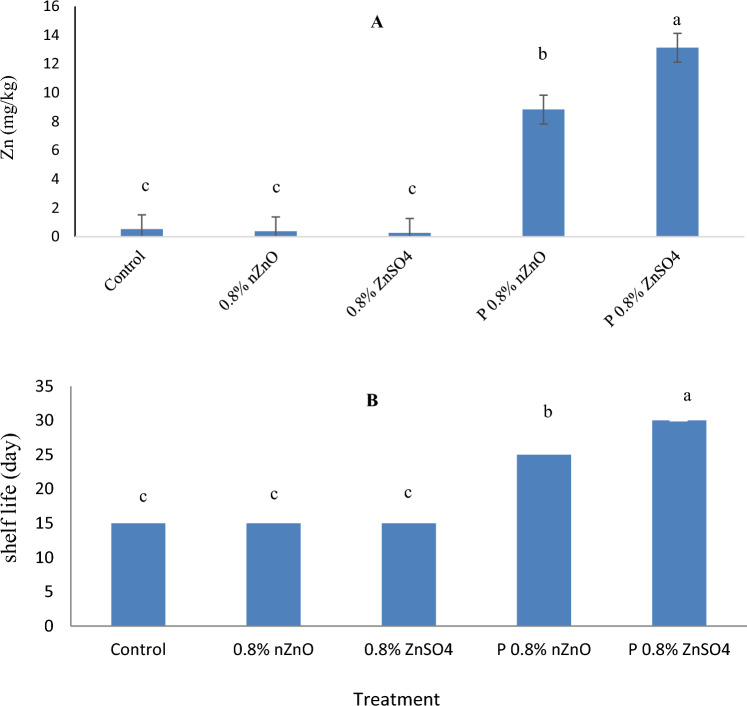
Zinc content (**A**) and Storage life (**B**) in arils treated with two forms of zinc including nano zinc oxide (nZnO) and zinc sulfate (ZnSO_4_) before and after harvest during storage at 5 °C. (P means postharvest).

Different treatments had various effects on the zinc content in arils (P ≤ 0.01). Arils treated with the postharvest 0.8%W/V ZnSO_4_ had the highest zinc content, which was substantially greater compared to the other treated arils. Arils of the control had the least quantity of zinc. Zinc uptake was higher in postharvest treatments because the exogenous zinc came into direct contact with the aril surface (Fig. 1a). In this research, the amount of zinc in pre-harvest treatment was lower than 0.5 mg/Kg, which increased by about 27 times in arils of the postharvest 0.8% W/V ZnSO_4_ treatment (Fig. 1a.). The allowable dose of zinc is 40 mg per day; however, this limit does not apply to people who suffer from zinc deficiency. The recommended daily allowance (RDA) is 8 mg in adult women, 11 mg in adult men and pregnant women, and 12 mg in lactating women. Furthermore, only 4 mg of this element is required in the diet of individuals aged 6 months or younger^24^. The amount of zinc in all treated arils did not exceed these limits of daily-allowance (LDA). The RDA of zinc can be met by fortified pomegranate arils. Also, according to the American Academy of Sciences, the maximum RDA of zinc per 100 g of food is 2.2 mg (20% of daily requirement)^25^. According to Fig. 1a. the maximum amount of zinc in arils (13.1 mg/kg) was measured in response to the 0.8% W/V ZnSO_4_ treatment after harvest, which is in accordance with this standard and did not exceed the RDA. Therefore, if about 170 gr of arils is consumed from the source of postharvest 0.8% W/V ZnSO_4_, it will be enough to meet the daily human need for zinc. Higher concentrations of zinc were associated with greater antibacterial effects (r = − 0.96)^26^. The results indicated that the Zn concentration with a coefficient of determination R^2^ = 91 alone justified most of the regression change. The above regression model with the coefficient of explanation R^2^ = 98 optimally justified the most regression changes. The path analysis (Table 1) of traits entering the regression equation indicated that Zn had the most direct effect on shelf life. The Zn treatment had the most direct effect (1.26) on the shelf life by limiting the activity of total aerobic microorganisms (Table 1).

### Storage life

Senescence in arils generally involves an increase in decay incidence, pH, PPO, and decreased TSS, TA, weight loss, phenol, anthocyanin, and antioxidant activity. Arils of the control and those treated in the pre-harvest stage deteriorated after fifteen days of storage. Samples of the postharvest 0.8% W/V nZnO treatment groups decayed after 25 days of storage. Arils of the postharvest 0.8% W/V ZnSO4 did not decay until the thirtieth day of storage (Fig. 1b.) and maintained the physicochemical values of arils better than the other treatments. Compared to the control group and the pre-harvest treatment groups, the postharvest treatment doubled the length of storage life. Zinc treatments significantly extended the storage life of pomegranate arils. The zinc content and storage life correlated significantly (r = 0.95) (Table 2). Thakur et al. (2021)^27^ stated that zinc increased the shelf life of crops, which confirms the results of the current experiment. The postharvest treatment resulted in a longer shelf life than the pre-harvest treatment.

**Table 2:** Pearson’s correlation coefficients in arils treated with different concentrations of two forms of zinc including nano zinc oxide (nZnO) and zinc sulfate (ZnSO_4_) at the end of the storage life at 5°C.

	WL	pH	TSS	TA	TPC	Anthocyanin	Antioxidant	PPO	T aerobic count	Psychotropic	T yeast and mold	Zn	Shelf life
WL	1												
pH	0.64*	1											
TSS	− 0.47 ns	− 0.77**	1										
TA	− 0.6**	− 0.86**	0.81**	1									
TPC	0.42 ns	0.55**	− 0.38 ns	0.76**	1								
Anthocyanin	0.6**	− 0.55**	0.78**	0.79**	− 0.4 ns	1							
Antioxidant	− 0.57**	− 0.57**	0.82**	0.80**	− 0.92 **	0.90**	1						
PPO	0.41 ns	0.58**	− 0.56**	− 0.70**	− 0.68**	− 0.60**	− 0.62**	1					
T aerobic count	0.56**	− 0.54**	− 0.72**	− 0.75**	0.88 **	0.89**	0.92**	− 0.72**	1				
Psychotropic	0.59**	− 0.76**	− 0.73**	− 0.85**	− 0. 88 **	0.82**	0.87**	0.81*	0.91**	1			
T yeast, mold	0.61*	− 0.61**	− 0.72**	− 0.75**	− 0.32 ns	0.78**	0.84**	0.79**	0.99**	0.84**	1		
Zn	− 0.52**	− 0.95**	0.75**	0.94**	− 0.30 ns	0.98**	0.94**	− 0.82**	− 0.97**	− 0.96**	− 0.96**	1	
Shelf life	− 0.56*	− 0.94**	0.81**	0.98**	− 0.39 ns	0.97**	0.97**	− 0.83**	− 0.91**	− 0.91**	− 0.91**	0.95**	1

* and ** means significant at 5% and 1% level of probability, respectively.

### Weight loss

**Figure 2:**
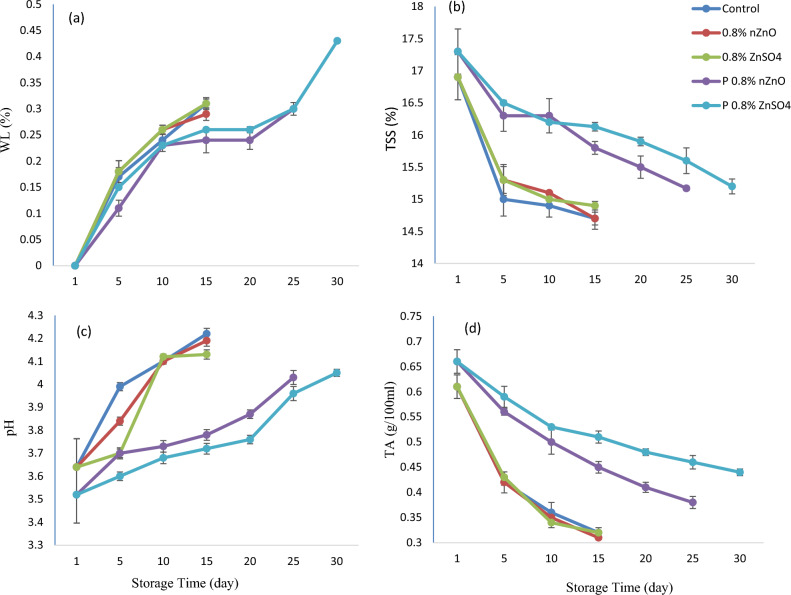
Changes WL (**a**), TSS (**b**), pH (**c**), and TA (**d**) in arils treated with two forms of zinc including nano zinc oxide (nZnO) and zinc sulfate (ZnSO_4_) before and after harvest method during storage at 5 °C. (P means postharvest).

WL intensified through the storage period, but was lowered in the postharvest zinc-treated arils (P ≤ 0.01) (Fig. 2a). WL was most intense in arils of the control group and in those treated with the pre-harvest method. The postharvest 0.8% W/V ZnSO_4_ treatment maintained the aril moisture optimally until the thirtieth day of storage. Pre-harvest treatment with zinc had no effect on WL during storage. In the postharvest treatment, however, zinc sulfate was more effective than nZnO. The storage of arils was associated with an exchange of moisture from within the aril to the outer environment, accompanied by tissue-breakdown and cellular respiration^1,28,29^. These results confirm previous studies that postharvest zinc treatments reduced WL in kiwifruit^30^, strawberry^14^ and pomegranate^15,31^. WL was reduced by zinc because of its effect on membrane integrity and microbial load (r = 0.6) (Table 2), accompanied by increasing the antioxidant activity because the zinc uptake was higher in the postharvest treatment.

### TSS, juice pH, and TA

During storage, the aril juice was characterized by an increase in pH, although it increased less markedly in the postharvest treatment group (P ≤ 0.01) (Fig. 2c). After fifteen days of storage, arils of the control and pre-harvest treatment groups had pH values higher than those of the postharvest treatment group. The minimal pH value occurred as a result of the postharvest 0.8% W/V ZnSO_4_ treatment on the thirtieth day of storage. Changes in the pH had a negative correlation with the TA (r = − 0.96). The zinc treatment ameliorated the decrease in TSS and TA, thereby slowing down the rise in pH value (r = 0.94) (Table 2). TSS and TA levels were highest at harvest and gradually decreased through storage (Fig. 2). Nonetheless, the least amount of decrease was observed in arils treated with the postharvest method. When compared to the control and the pre-harvest method, arils of the postharvest treatment group had more TA and TSS values during storage. TSS and TA became highest in response to the postharvest 0.8% W/V ZnSO_4_. Meanwhile, the control group was characterized by the least amount of TA and TSS. Furthermore, the pre-harvest zinc treatment had no effect on pH, TA, and TSS at the time of storage. Regarding the postharvest group, the zinc sulfate treatment was more effective than the nZnO. The zinc treatment effectively reduced the TSS and TA during storage which could reduce the microbial load (r = − 0.96)^32^. Since energy consumption, respiration, and fermentation negatively affect the amount of organic acids, the results of this research confirmed those of a previous report on strawberry in which the microbial contamination resulted in a shorter storage life^33^. Since organic acids are less changed when a lack of oxygen persists, the decrease in TA could be linked to their oxidation^18^. Zinc treatment reduced the rate of microbial contamination and reduced the declining trend of TSS and TA factors. Organic acids contribute signficantly to the synthesis of phenolic compounds through the provision of a carbon structure^34^. Decreased TSS in the latter stages could result from carbohydrate consumption^35^. TA is usually defined in association with organic acids. The decrease of acidity occurs when organic acids are consumed in respiration^36^. In a relevant study, an aqueous solution of zinc sulfate (1%) was sprayed on mango leaves once or twice, at 20-day intervals in May, and then the fruits were stored in cold storage after harvest. The results showed that the samples of the two-time pre-harvest foliar spray improved the TSS and better maintained fruit quality^37^.

### Anthocyanin content

**Figure 3:**
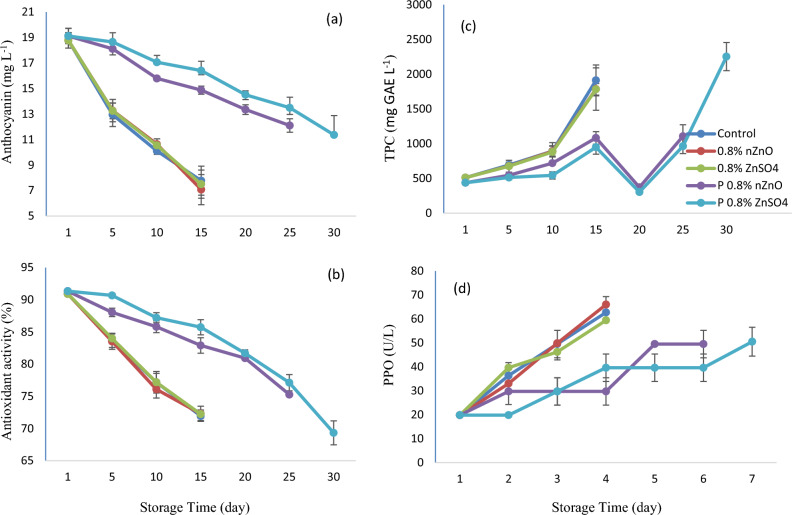
Changes Anthocyanin (**a**), Antioxidant Activity (**b**), TPC (**c**) and PPO enzyme (**d**) in arils treated with two forms of zinc including nano zinc oxide (nZnO) and zinc sulfate (ZnSO_4_) before and after harvest method during storage at 5 °C. (P means postharvest).

The color of pomegranates is determined by their anthocyanin content, which includes delphinidin, pelargonidin, and cyanidin^38^. There was a decrease in anthocyanin content through the 30 days of storage in this study. A slower rate of this decrease was observed in the postharvest zinc treatment group, compared to the control and the pre-harvest group (P ≤ 0.01) (Fig. 3a). The control group and the pre-harvest group had the lowest anthocyanin content, whereas the postharvest 0.8% W/V ZnSO_4_ had significantly the highest. The postharvest 0.8% W/V ZnSO_4_ treatment had a greater effect on retaining total anthocyanin content than the other treatments, and zinc sulfate was more effective than nZnO in this regard. The oxidation of TPC catalyzed by PPO is a precursor of anthocyanin loss in storage^14,39^.

### Antioxidant activity

Antioxidant activity was reduced in all treatment groups during storage; however, the reduction rate was lower in arils treated with the postharvest zinc, compared to the control (P ≤ 0.01), (Fig. 3b). Non-treated arils and those treated by pre-harvest method after 15 days of storage had the lowest antioxidant activity. Nonetheless, arils treated with the postharvest 0.8% W/V ZnSO_4_ showed the highest antioxidant activity at this stage and differed substantially from the other treatment groups. In other words, the pre-harvest treatment with zinc did not contribute to the antioxidant activity retention in storage, whereas in the postharvest treatment, zinc sulfate was more effective than the nZnO. The antioxidant activity declined in storage, which could be linked to gradual decay. The postharvest zinc treatment ameliorated the reduction of antioxidant compounds (r = 0.94), similar to previous research^14,15,40^. Also, Emamifar et al. (2010)^7^ stated that adding nano ZnO to the coating formulation enhanced the antioxidant and antimicrobial activities. Zinc increases binding capacity and antioxidant function. It decreases cellular oxidative stress by contributing to mitochondrial homeostasis^37^. An experiment by Deng et al. (2020)^41^ showed that the short-term exposure of jujube fruits to a solution of mineral ions such as zinc, copper, and iron delayed the aging of jujube fruits after harvest, thereby improving its nutrient quality, which is attributable to a longer fruit shelf life as a result of the rise in NADPH oxidase (NOX), which led to a higher level of antioxidant biosynthesis, decreased free radicals of oxygen (ROS), and delayed cellular aging.

### Total phenols content

TPC increased during the initial 15 days and then decreased until day 20. Thereafter, the TPC increased again until the storage period ended (Fig. 3c). The TPC in arils of the postharvest zinc treatment was higher than that of the control and the pre-harvest treatment group (P ≤ 0.01). Throughout the sampling time, arils of the postharvest 0.8% W/V zinc-sulfate treatment had maximum levels of TPC, whereas the control and the pre-harvest group had the lowest. In fact, the pre-harvest treatment of zinc did not benefit phenol retention during storage. The postharvest treatment became more efficient in response to higher zinc concentrations, but the zinc sulfate was more effective than the nZnO. Increasing the ZnO concentration to 1.25 g/L reportedly increased the TPC retention, compared to the control^7^, which is similar to the results of this experiment in the postharvest treatment. The increase in TPC throughout storage could manifest higher biosynthetic enzyme activity in cold storage^42^, or cellular aging due to cellular breakdown^17^. TPC reportedly decreased because of enzymatic activity^43^, which could be attributed to TPC oxidation, polymerization, or cellular breakdown during aging^44^. As a major cause of fruit browning, the PPO activity reduces the TPC. The increase in the PPO activity in browned arils leads to a relative reduction of the phenol content. TPC enzymatic oxidation, resulting in aril browning, could be attributed to PPO activity. This could be attributed to significantly reactive o-quinones that form brown pigments and spoil the natural color of fruits^45^. Zinc-treated arils could also prevent the decrease in phenols by reducing the microbial population and withstanding cellular breakdown, compared to the control group^15^.

### Polyphenol oxidase activity

During storage, the PPO activity increased more in the control and the pre-harvest group, compared to the postharvest treatment (P ≤ 0.01), (Fig. 3d). Arils of the control and those treated at the pre-harvest stage had the highest PPO activity, whereas the least was observed in the postharvest 0.8% W/V ZnSO_4_ which was significantly different from the other treatment groups. In fact, the efficiency of the postharvest treatment increased in response to higher zinc concentrations, and, among the treatments, the zinc sulfate was more effective than the nZnO. The main activity of PPO is between pH 4 and 7, and the optimum activity of PPO is observed at pH 7. It has been reported that even small changes in tissue pH can greatly affect PPO activity and thus browning rate. Membrane stability is an important factor in controlling color changes. Zinc plays a role in maintaining the structure and function of biological membranes^46^. A gradual rise in the activity of PPO could account for the quick browning of arils. As the PPO is mobile within thylakoid membranes, it affects the cytoplasmic layer and causes browning^21^. In the present research, the brown arils had high pH values. Their moisture content was lower in comparison with that of the healthy ones. Given such changes, imbalance is created between oxidative and reductive processes, thereby decreasing membrane integrity. This could also make the TPC enzymatic oxidation process easier until brown polymers are formed^45^. Sulfates and vitamin C usually operate at active sites of enzymes and inhibit PPO activity^47^.

### Microbial evaluation

**Figure 4:**
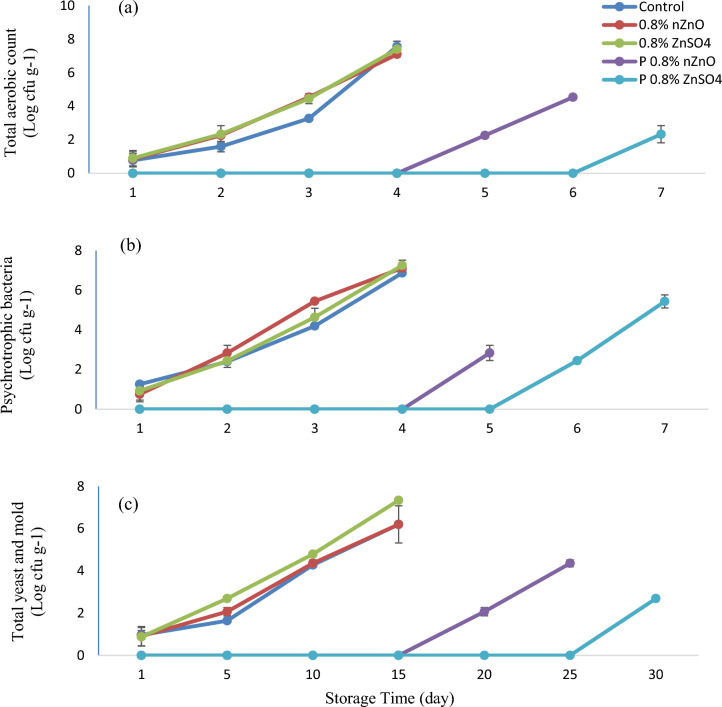
Total aerobic count (**a**), psychrotrophic bacteria (**b**), and total yeast and mold (**c**) in arils treated with two forms of zinc including nano zinc oxide (nZnO) and zinc sulfate (ZnSO_4_) before and after harvest during storage at 5 °C.

**Figure 5:**
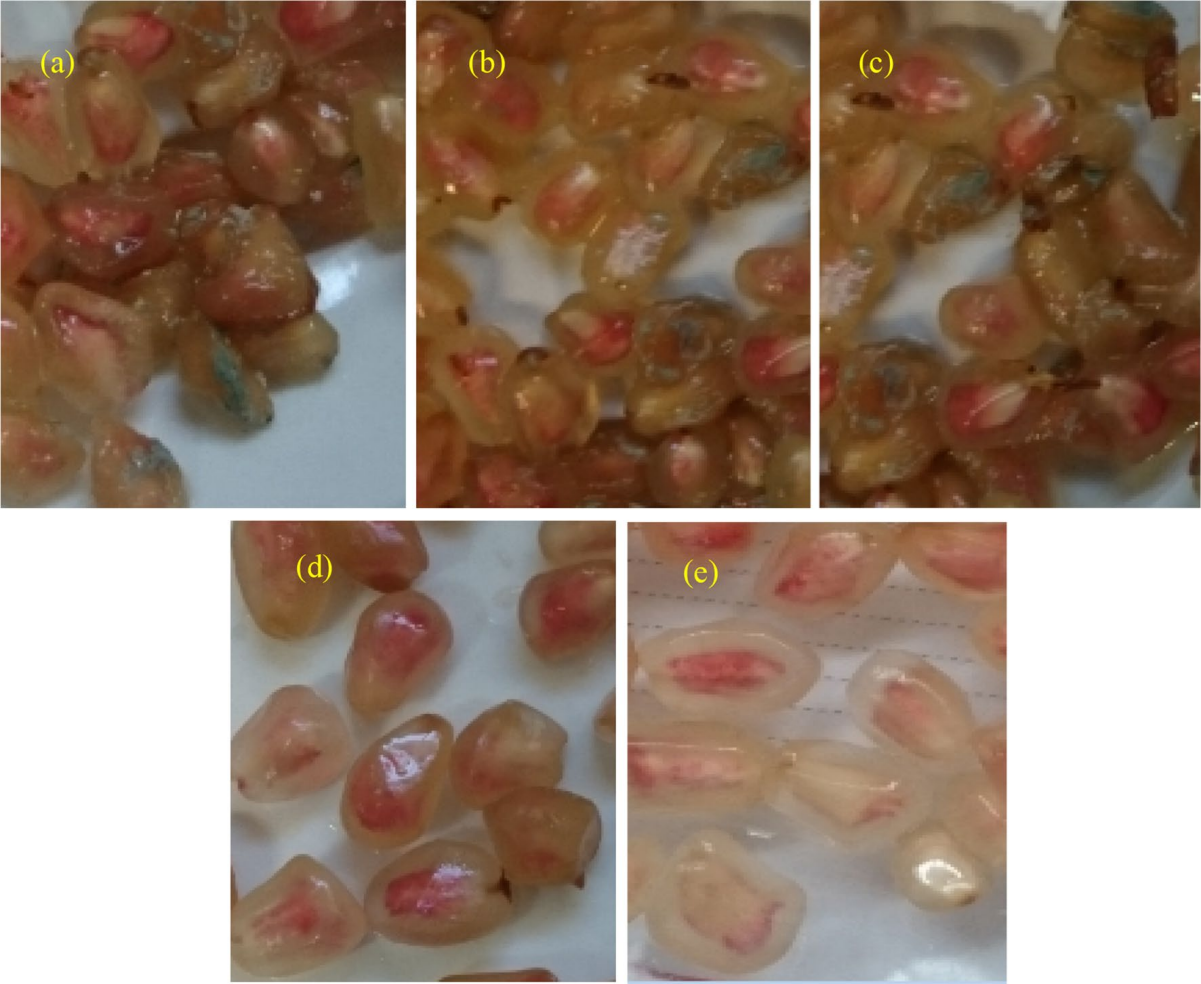
Visual quality of arils during storage a: control (pre harvest) at the end of the storage life (15th day), b: pre harvest nZnO 0.8% W/V at the end of the storage life (15th day), c: pre harvest ZnSO_4_ 0.8% W/V at the end of storage life (15th day). d: postharvest nZnO 0.8% W/V at the end of the storage life (25th day), e: postharvest ZnSO_4_ 0.8% at the end of storage life (30th day).

The microbiological properties of pomegranate arils (Fig. 4) changed significantly during storage (P ≤ 0.01). Coliforms were not detected in the treated arils. The maximum allowable limits of total yeast, mold, and total aerobic microbes are 5 and 7 log CFU/g, respectively^22^. Total aerobic count in the control and the pre-harvest treatment increased to 7 log CFU/g on day fifteen but not in the samples of the postharvest treatment. The count of yeasts and molds exceeded the maximum limit on day fifteen and twenty of storage in the control and the pre-harvest stage, respectively. Therefore, the postharvest ZnSO_4_ and nZnO improved the hygienic conditions of arils. The postharvest treatment became more efficient when a higher zinc concentration was used. The postharvest ZnSO_4_ was more effective than the postharvest nZnO in terms of microbial preservation as well as visual quality of arils when the storage period ended (30 days) particularly in samples treated with 0.8% W/V ZnSO_4._ These findings were in good agreement with those of previous studies on pomegranate^15^ and strawberry^14^. The zinc treatment reduced the microbial growth and slowed the decaying process. It decreased mycelium growth and its impact on bacterial survival was dependent on the bacterial species^48^. The zinc treatment can inactivate the most essential proteins required for microbial survival. For example, degradation of Fe-S clusters may occur at high zinc concentration^49^. With the increase in storage life, the ROS increased, owing to physical damage, moisture loss, microbial attack, membrane fat oxidation, and cellular damage, ultimately causing cell death. However, cell death in plants is expected to decrease remarkably in response to higher concentrations of antifungal and antibacterial agents. Zinc antimicrobial activity could be attributed to the release of zinc ions, oxidative stress reduction due to ROS production, and irregularities in the plant cell wall, owing to zinc particle accumulation^7^. In the current research, the postharvest ZnSO_4_ treatment was more effective than zinc nanoparticles. ZnSO_4_, similar to zinc-acetate, can produce Zn^+2^ after dissolution in water. The ions become attached to microorganism membranes, thereby delaying the cell-division and growth cycle. The solubility of zinc-oxide nanoparticles in water is weak (0.0004 g/100 mL vs. 57.7 g/100 mL for ZnSO_4_); therefore, they do not have an even distribution^50^. The nZnO can affect microbial populations in ways that are not yet fully understandable. The assumption is that ROS accumulation causes a loss of uniformity in membrane components and then zinc-nanoparticles accumulate on bacterial membranes^51^. Interactions between zinc-oxide nanoparticles and bacteria can change cellular permeability through the provision of hydroxide ions (OH^−^), hydrogen-peroxide (H_2_O_2_) and superoxide ions (O_2_^−^). Thus, bacterial cell growth becomes impeded by oxidative-stress, sometimes leading to cell death^52^. Despite this, there is no report of a similar observation in human cells; therefore, it may have no adverse effect on health^15^. The decrease of decay could be partly due to the metals (e.g., Zn^+2^) which mean that ROS metabolism is aggravated by bacterial or fungal proliferation^7,41^. Therefore, in the postharvest treatment, since zinc comes into direct contact with the arils, it can reduce microbial contamination more effectively and increase the storage life to a greater extent, compared to the pre-harvest treatment, where zinc was sprayed on the plant. The arils changed in terms of visually perceivable features during storage (Fig. 5).

### Sensory evaluation

**Figure 6:**
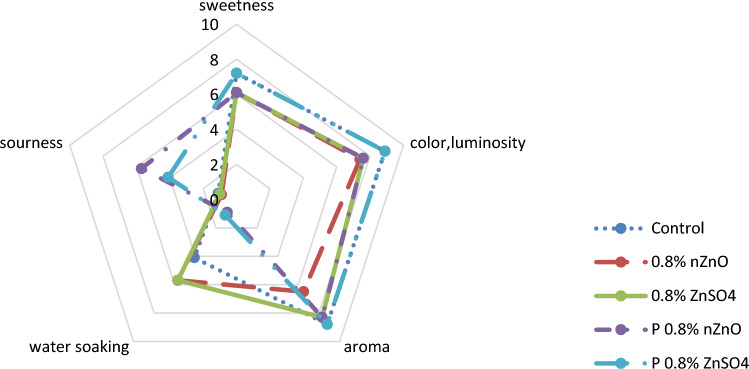
The average sensory scores in arils treated with two forms of zinc including pre-harveat and postharvest nano zinc oxide (nZnO) and zinc sulfate (ZnSO_4_) after 15 days of storage at 5 °C.

Arils of the control group and those of the pre-harvest treatment were characterized by lighter colors and weaker luminosity, fainter sweetness, less aroma and sourness, and more water-soaking, according to the panelists, whereas arils of the postharvest treatment scored better sensory values. The zinc concentration (both chemical forms) did not damage the aroma or luminosity of the arils. On day 15 of storage, the postharvest 0.8% W/V ZnSO_4_ caused the arils to be better in terms of sensory attributes, compared to the other treatment groups. Our findings also revealed that the postharvest zinc treatment controlled the extent of pigment-browning by limiting phenol oxidation (Fig. 5). The postharvest zinc treatment also decreased microbial growth during storage, which may have a positive effect on sweetness, aroma, sourness, and water-soaking of the arils (Fig. 6). Our findings are in line with those of previous research on strawberries^14^ and pomegranate^15^. The sensory evaluation improved by adding the postharvest zinc treatment on arils and became more effective at higher concentrations of Zn. In the post-harvest treatment, the zinc sulfate was more effective than the nZnO.

## Conclusion

Enriching arils with zinc in the postharvest stage maintained the quality and extended the storage life. The postharvest treatment was more effective than the pre-harvest treatment. The zinc treatment limited WL and PPO while slowing the decrease in anthocyanin content, antioxidant activity, TSS, and TA. In comparison with the control and the pre-harvest treatment, the postharvest treatment effectively preserved the visually perceived characteristics and nutritional qualities of arils. Among the postharvest treatments, the effect of 0.8% W/V ZnSO_4_ was most optimal, followed by 0.8% W/V nZnO, for maintaining aril quality during storage. The zinc concentration increased in the arils but did not exceed the allowable limit of daily intake. Thus, this approach can be considered safe for the bio-enrichment of zinc in arils. However, the intake of other zinc-sourced foods during the day cannot be avoided. Meat, dairy products, and fish are examples that are rich in zinc. Thus, care should be taken when eating zinc-enriched arils on the same days of eating other zinc-rich foods.

## Data Availability

The datasets used and/or analyzed during the current study available from the corresponding author on reasonable request.
